# WANDA: an end-to-end solution for tele-monitoring of chronic conditions

**Published:** 2012-06-15

**Authors:** Majid Sarrafzadeh, Richard Sykes

**Affiliations:** UCLA, USA; NetSCientific, UK

**Keywords:** remote monitoring, chronic disease, tiered architecture, analytics, clinical study

## Abstract

**Introduction:**

Approximately two-thirds of the deaths in the world are caused by chronic diseases such as cancer, diabetes, heart disease, and lung diseases. Chronic diseases are increasing in frequency and are costing the world $47 trillion in treatment costs and lost wages. Remote monitoring platforms promise to revolutionize healthcare by reducing healthcare costs and improving quality of care in chronic disease management. WANDA, designed at UCLA and deployed in clinical settings, is a three tiered, end-to-end remote patient monitoring solution with extensive hardware/software components designed to cover the broad spectrum of the telehealth paradigm. The system tiers consist of a data collection framework, a data storage and management, and an analytics engine, all supported by several applications for data visualization, annotation, and social support.

**Aims and objectives:**

Our primary objective was to build basic elements of WANDA that enables deployment of the system in clinical settings and test feasibility and utilization of the system in monitoring chronic conditions. To this end, we have developed user-friendly hardware/software elements for data collection, regulatory compliant data storage, and highly flexible and adaptable data analytics which facilitate physician data review and provide built-in ability to identify statistically significant correlations between phenomena, conditions, and medical events for each defined chronic disease, and predicting clinical episodes and medical complications such as hospitalizations, heart attacks, asthma attacks, and diabetes complications so that such events can be mitigated, reducing care costs and improving patient quality of life.

**Methods:**

A data collection framework was designed to acquire data from a set of heterogeneous off-the-shelf sensor nodes including blood pressure monitors, blood glucose monitors, weight scales, pulse oximeters, and accelerometers, and transmit the data, through an Android phone, to a back-end server. Multivariate data imputation techniques were used to estimate any missing values in the continuously collected data. Statistical egression techniques were applied to the medical signals to identify statistically significant correlations between events of interest. A feature selection algorithm was developed to improve the regression methods. The system has been used in five clinical studies including three heart failure studies, a diabetes study, and a weight loss study. The system is currently used in a large clinical study with a total of 1500 heart failure patients are being recruited from six sites across California. In a smaller study in the past, we have recruited a total of 21 patients (10 men and 11 women; mean age 73.1±9.3, range 58–88) who completed a three-month intervention. A reference group of 21 patients (matched on age, gender, and race) was included in the preliminary analysis to provide a broad comparison group for studying patients. Baseline socio-demographic and clinical characteristics of the two groups were comparable. Both groups showed improvements in perceived health, HRQOL, and depression over time; however, anxiety increased in the comparison group. Patients assigned to the intervention group showed greater improvements in all six psychosocial factors over time.

**Results:**

Positive patient receptiveness and attitude toward using WANDA, and our preliminary results on improvements made by patients in the psychosocial factors are promising. Our data imputation approach, applied on predicting missing answers for 12 daily questionnaires shows an accuracy of 83%. Our feature selection algorithm finds optimal feature set (20 features) usable for regression analysis while maintaining the same accuracy as the full feature set.

**Conclusions:**

The WANDA is built on a three-tier architecture. The first tier is a sensor tier that measures patients’ vital signals and transmits data to the web server tier. The second tier consists of web servers that receive data from the first tier and maintains data integrity. The third tier is a back-end database server and analytics engine.

